# Effects of Cardiorespiratory Fitness on Cerebral Oxygenation in Healthy Adults: A Systematic Review

**DOI:** 10.3389/fphys.2022.838450

**Published:** 2022-03-04

**Authors:** Talia Salzman, Olivier Dupuy, Sarah Anne Fraser

**Affiliations:** ^1^Faculty of Health Sciences, School of Human Kinetics, University of Ottawa, Ottawa, ON, Canada; ^2^Laboratory MOVE, University of Poitiers, Poitiers, France; ^3^Faculty of Medicine, School of Kinesiology and Physical Activity Sciences (EKSAP), University of Montreal, Montreal, QC, Canada; ^4^Faculty of Health Sciences, Interdisciplinary School of Health Sciences, University of Ottawa, Ottawa, ON, Canada

**Keywords:** cardiorespiratory fitness, functional near-infrared spectroscopy, cerebral oxygenation, younger adults, older adults, exercise, cognition

## Abstract

**Introduction:**

Exercise is known to improve cognitive functioning and the cardiorespiratory hypothesis suggests that this is due to the relationship between cardiorespiratory fitness (CRF) level and cerebral oxygenation. The purpose of this systematic review is to consolidate findings from functional near-infrared spectroscopy (fNIRS) studies that examined the effect of CRF level on cerebral oxygenation during exercise and cognitive tasks.

**Methods:**

Medline, Embase, SPORTDiscus, and Web of Science were systematically searched. Studies categorizing CRF level using direct or estimated measures of V̇O_2*max*_ and studies measuring cerebral oxygenation using oxyhemoglobin ([HbO_2_]) and deoxyhemoglobin ([HHb]) were included. Healthy young, middle-aged, and older adults were included whereas patient populations and people with neurological disorders were excluded.

**Results:**

Following PRISMA guidelines, 14 studies were retained following abstract and full-text screening. Cycle ergometer or treadmill tests were used as direct measures of CRF, and one study provided an estimated value using a questionnaire. Seven studies examined the effects of CRF on cerebral oxygenation during exercise and the remaining seven evaluated it during cognitive tasks. Increased [HbO_2_] in the prefrontal cortex (PFC) was observed during cognitive tasks in higher compared to lower fit individuals. Only one study demonstrated increased [HHb] in the higher fit group. Exercise at submaximal intensities revealed increased [HbO_2_] in the PFC in higher compared to lower fit groups. Greater PFC [HHb] was also observed in long- vs. short-term trained males but not in females. Primary motor cortex (M1) activation did not differ between groups during a static handgrip test but [HHb] increased beyond maximal intensity in a lower compared to higher fit group.

**Conclusion:**

Consistent with the cardiorespiratory hypothesis, higher fit young, middle-aged, and older adults demonstrated increased cerebral oxygenation compared to lower fit groups. Future research should implement randomized controlled trials to evaluate the effectiveness of interventions that improve CRF and cerebral oxygenation longitudinally.

## Introduction

Considerable evidence has supported that maintaining a good level of physical activity is associated with better cognitive performances across the lifespan (Colcombe and Kramer, 2003; Hillman et al., 2008; Stillman et al., 2020). Themanson et al. (2006) suggest that such effects may be driven by exercise-related improvements in cardiorespiratory fitness (CRF). From childhood to adulthood, a greater CRF will have a positive impact on cognitive performance. More specifically, executive performance seems to be preferentially favored. Indeed, there is considerable evidence from cross-sectional studies and meta-analyses that CRF has beneficial effects on multiple cognitive domains, particularly executive functions (Colcombe and Kramer, 2003; Predovan et al., 2012; Dupuy et al., 2015; Chaparro et al., 2019; Goenarjo et al., 2020a). This is partly because the prefrontal cortex, which governs these functions, seems to be very sensitive to physical activity-related changes (Yuki et al., 2012). Therefore, the interactions between exercise and cognition are multifaceted and examining them requires a deeper understanding of cognitive and physiological concepts.

The clinical benefits of CRF on cognitive function appear in the form of enhanced brain functioning, as suggested by neuroimaging studies that report increased brain activity in physically active older adults when compared to less active ones (Voelcker-Rehage and Niemann, 2013). Evidence from functional magnetic resonance imaging (fMRI) studies also supports the effects of CRF on brain activation during cognitive tasks. For instance, active older adults exhibited increased brain activity in the prefrontal cortex (PFC) and decreased activity in the anterior cingulate cortex compared to less active older adults during Flanker and Stroop tasks (Colcombe et al., 2004). This is consistent with the cardiorespiratory hypothesis which suggests that higher levels of fitness are related to increased cerebral blood flow (Agbangla et al., 2019b).

Many underlying neurophysiologic and structural changes may be used to explain this improvement in brain functioning (Hillman et al., 2008; Ploughman, 2008). For example, structural brain changes after physical training including both the improvement of the density and integrity of gray and white matter has been observed (Weinstein et al., 2012; Voelcker-Rehage and Niemann, 2013; Erickson et al., 2014; Sexton et al., 2016; d’Arbeloff, 2020; Kundu et al., 2021). However, it should be noted that the functional activity of the brain is related to blood supply and that neuronal activation requires an increase in cerebral blood flow and metabolism. This mechanism has been illustrated by Mehagnoul-Schipper et al. (2002) who reported simultaneous increases in cerebral blood volume and cerebral oxygenation during motor tasks in younger and older adults using functional near-infrared spectroscopy (fNIRS) and fMRI. It also appears that the magnitude of this adaptation is proportional to the complexity of the task. Several studies have reported that during exercise (Mekari et al., 2015) or by using hypoxic paradigms (Ando et al., 2013; Williams et al., 2019), cerebral oxygen availability affects cognitive performance and shows that cerebral oxygenation plays a key role in brain functioning.

Among neuroimaging techniques, however, fMRI has limited applications during exercise tasks that involve a full range of motion. FNIRS can overcome these challenges since it is portable, non-invasive, and robust to motion artifacts, which is also convenient for examining participants of all ages (Quaresima and Ferrari, 2016). Both fMRI and fNIRS function based on the principles of neurovascular coupling but fNIRS dissociates oxyhemoglobin ([HbO_2_]) and deoxyhemoglobin ([HHb]) unlike the blood oxygen level dependent (BOLD) response measure used in fMRI (Villringer, 1997). This is because fNIRS devices emit near-infrared light into the scalp at specific wavelengths (i.e., 650–950 nm) that coincide with the absorption properties of [HbO_2_] and [HHb] (Scholkmann et al., 2014). The reflected light is then measured by continuous wave, frequency domain, or time domain techniques, which differ based on how the incident light is emitted and reflection is detected. Continuous wave devices, which are the most commercially available, rely on a constant intensity of light and quantify the relative changes in reflected light (Scholkmann et al., 2014). In contrast, frequency and time domain devices are more complex but capture absolute measures of cerebral oxygenation. Frequency domain devices modulate the incident light and measure the phase shift of the reflected light compared to time domain devices that emit short pulses of light and measure the dispersion of reflected light (Scholkmann et al., 2014). Lastly, fNIRS also has the advantage of better temporal and spatial resolution than fMRI and electroencephalography (EEG), respectively, and at relatively a low cost (Pinti et al., 2018). For these reasons, fNIRS publications have grown exponentially with many studies focusing on exercise and cognition (Yan et al., 2020). However, few reviews focus on the role of CRF on cerebral oxygenation during cognitive or physical activity and to our knowledge none have examined this systematically (Agbangla et al., 2021).

One factor that increases heterogeneity amongst reviewed studies is the assessment of CRF. The gold standard index of CRF, V̇O_2*max*_, reflects a point where an individual’s maximum oxygen uptake remains constant despite an increase in workload (Buttar et al., 2019). It also provides the most accurate assessment of CRF and can be evaluated using direct or estimated measures (Vanhees et al., 2005; Aadahl et al., 2007). Direct measures typically involve running or cycling tests with incremental loads (Vanhees et al., 2005). Therefore, direct measures are generally assessed in a laboratory setting using cardiopulmonary exercise systems and gas analyzers that are worn during the test and capture ventilatory measurements, oxygen consumption, and expired carbon monoxide. Since direct measures require specialized equipment and settings, a valid alternative is to use maximal or submaximal tests that require minimal equipment or self-report questionnaires that estimate CRF (i.e., V̇O_2*max*_) using an equation. For example, indirect tests include the Rockport test (Jurca et al., 2005; Dupuy et al., 2018), Balke test (Balke and Ware, 1959; Goenarjo et al., 2020b,^2021^), or 1.5 mile (George et al., 1993) run where participants may or may not be equipped with heart rate monitors. Accordingly, aerobic capacity may be estimated based on heart rate, distance covered, or trial time using the validated protocols for each type of test (George et al., 1993; Buttar et al., 2019). Lastly, self-report cardiorespiratory measures have been shown to correlate with V̇O_2*max*_ but risk leading to overestimations of fitness levels in sedentary people (Aadahl et al., 2007).

The present review aims to consolidate findings from previous reports that demonstrated an interaction between CRF level and cerebral oxygenation. More specifically, this systematic review will summarize evidence from cross-sectional and interventional fNIRS studies that used direct or estimated measures of CRF and examined changes in cerebral oxygenation in healthy younger, middle-aged, and older adult groups during cognitive and exercise tasks. Findings will provide insights into the physiological mechanisms underlying changes in brain activation that are associated with CRF.

## Methods

This systematic review followed the Preferred Reporting Items for Systematic Reviews and Meta-Analyses (PRISMA) guidelines (Liberati et al., 2009).

### Eligibility Criteria

The PICO (population, intervention, comparison, and outcome) model was used to outline the inclusion and exclusion criteria for this systematic review (Eriksen and Frandsen, 2018). The population being studied included healthy younger, middle-aged, and older adults (i.e., participants aged 18 years and older). As this review focuses on healthy adults, studies that examined participants who are obese or patient populations including those with chronic neurological disorders were excluded. Intervention type included cognitive, physical, or a combination of both while fNIRS was used to assess changes in cerebral oxygenation during task performance. The studies must have also compared a high- and low-fit group or an active and control group that were created based on direct or estimated measures of CRF. Cross-sectional and longitudinal studies were included whereas those without full-texts, systematic reviews, and animal studies were excluded.

### Search Strategy

Following PRISMA guidelines, the systematic literature search was conducted in July 2021 using four online databases: Medline, Embase, SPORTDiscus, and Web of Science. There were no restrictions set for language or publication year. The search terms encompassed Medical Subject Headings (MeSH) and keywords that described fNIRS, the hemodynamic response as well as CRF levels (Supplementary Table 1). For example, the following keywords and MeSH terms were used: “Spectroscopy, Near-Infrared” AND “Cardiorespiratory fitness” OR “V̇O_2*max*_” AND “[HbO_2_]” OR “[HHb].”

The resulting studies were imported and managed in Covidence (Melbourne, Australia) where duplicates were automatically removed and visually inspected by TS. Titles and abstracts were then independently screened by OD and TS. SF resolved all conflicts and relevant studies were retained for the full-text review. SF, TS, and OD reviewed the full-texts and disagreements were resolved by a consensus between authors. The reference lists of the included full-texts were hand-searched as well as recent publications since the initial search for additional studies meeting the inclusion and exclusion criteria.

### Quality Assessment

The Joanna Briggs Institute (JBI) checklist for analytical cross-sectional studies was used to assess the methodological quality of the included studies (Ma et al., 2020). It is suitable for non-randomized experimental studies because it evaluates eight criteria: (1) Inclusion criteria in the sample were clearly defined; (2) The study subjects and the setting were described in detail; (3) The exposure was measured in a valid and reliable way; (4) Objective, standard criteria were used for measurement of the condition; (5) Confounding factors were identified; (6) Strategies to deal with confounding factors were stated; (7) The outcomes were measured in a valid and reliable way; and (8) An appropriate statistical analysis was used. The overall appraisal to determine whether a study is of sufficient quality to include, exclude, or seek further information, was decided based on a consensus between TS and SF.

### Synthesis of Results

Data extraction was conducted by TS and SF. The form included information on study and participant characteristics, study design, CRF measures, and fNIRS measures. Significant differences between CRF scores were identified across both direct and estimated measures. The hemodynamic response was extracted based on the study’s primary outcome measure. In addition, the type of task during which the hemodynamic response was measured (e.g., cycling, Stroop task) was analyzed while accounting for variables such as age, sex, and exercise intensity.

## Results

### Study Selection and Characteristics

**FIGURE 1:**
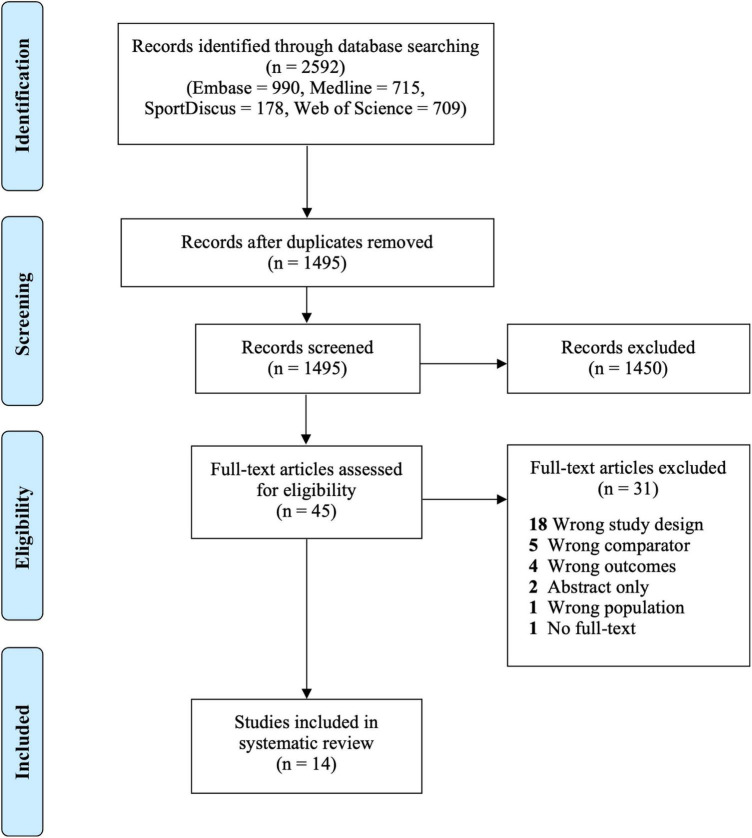
PRISMA flow diagram.

The initial database search resulted in 2,592 studies and 1,495 after duplicates were removed. After screening the titles and abstracts, 1,450 records were excluded. The full-texts of 45 studies were then reviewed and 31 studies were excluded due to the wrong study design (*n* = 18), wrong comparator (*n* = 5), wrong outcomes (*n* = 4), abstract only (*n* = 2), wrong population (*n* = 1), and no full-text available (*n* = 1) (Figure 1). No additional papers were included following a hand-search. Fourteen studies were retained for analysis. The publication period ranged from 2010 to 2020 and all the studies were cross-sectional except for one which was a pre-/post-intervention design. Four studies took place in France, followed by three in Japan, two in Australia and Canada, and one in Belgium, Germany, and the United States, respectively. Study characteristics for all 14 included studies are shown in Table 1.

**TABLE 1:** Study characteristics of included studies.

Author (Year)	Study design	Brain region	Task	Cerebral oxygenation outcomes
**Cognitive**				
Agbangla et al. (2019a)	Cross-sectional	PFC	N-back (0–3 back)	Bilateral increase [HbO_2_] in PFC during 2-back (*p* < 0.05) and 3-back (*p* < 0.05) compared to 1-back condition in high fit group. Decreased [HbO_2_] overall in low compared to high fit group (*ps* < 0.05). Greater decrease in [HHb] in the left hemisphere in high compared to low fit group (*p* = 0.003).
Albinet et al. (2014)	Cross-sectional	DLPFC	Control counting task and Random Number Generation at a fast (one tone every 1 s) or slow pace (one tone every 1.5 s)	Increased [HbO_2_] in left and right DLPFC during the Random Number Generation task in high fit group (*p* < 0.05). Decreased [HbO_2_] in right DLPFC in low compared to high fit group (*p* < 0.05). Decreased [HbO_2_] in left compared to right DLPFC in low fit group (*p* < 0.05). No change in [HbO_2_] or [HHb] between groups during the counting number task (*ps* > 0.3).
Dupuy et al. (2015)	Cross-sectional	DLPFC, VLPFC,	Computerized Stroop task	Greater increase in [HbO_2_] right inferior frontal gyrus of the VLPFC in high fit group compared to low fit (*p* < 0.01).
Hyodo et al. (2016)	Cross-sectional	DLPFC	Color-word Stroop task	Left lateralized DLPFC activation in high compared to low fit group. No differences in [HHb] *(p* > 0.05).
Mekari et al. (2019)	Cross-sectional	Left PFC	Trail making test A and B	Cerebral oxygenation mediated the relationship between cardiorespiratory fitness and Trail Making Test part B performance in the higher fit group (*p* = 0.004).
**Dual-task**				
Goenarjo et al. (2020a)	Cross-sectional	PFC	Auditory n-back (2-back) and walking	Greater decrease in [HHb] in the right and left PFC in high compared to low fit group (*p* = 0.01). Negative correlation between [HHb] and V̇O_2 peak_ (*r* = −0.36, *p* = 0.04). No differences in [HbO_2_].
**Exercise**				
Brugniaux et al. (2014)	Cross-sectional	Left PFC	Incremental cycling test	Increased [HbO_2_] from low to moderate intensity exercise in the active group compared to sedentary group (*p* < 0.05).
Buzza et al. (2016)	Cross-sectional	Left PFC	Ramp incremental peak and submaximal square wave cycling	No differences during ramp incremental task (*p* = 0.773) or submaximal square wave cycling (*p* = 0.788) in [HHb] between long and short term trained individuals.
Buzza et al. (2020)	Cross-sectional	Left PFC	Ramp incremental peak and submaximal square wave cycling	Increased [HHb] in PFC at 90% GET during submaximal square wave cycling (*p* = 0.011) in long compared to short term trained individuals.
Caen et al. (2019)	Pre-/post-intervention	Right PFC	6-week incremental ramp exercise	Steeper [HbO_2_] (*p* = 0.034) and [HHb] (*p* < 0.001) and increased [HbO_2_] amplitude (*p* < 0.001) at moderate intensities post-intervention compared to pre-intervention.
Oussaidene et al. (2015)	Cross-sectional	Left PFC	Maximal ramp cycle exercise test and supramaximal test	Increased [HbO_2_] (*p* < 0.05) at submaximal intensity in endurance trained compared to untrained group. No differences in [HHb] between groups.
Seidel et al. (2019)	Cross-sectional	M1, PMC, SMA, SPL, IPL	Incremental cycling test and multi-intensity cycling test	No differences in [HbO_2_] (0.444 ≤ *p* adjusted ≤ 0.980) or [HHb] between groups during multi-intensity cycling test (0.462 ≤ *p adjusted* ≤ 0.993) between endurance athletes and active controls. Across exercise intensities, decreased [HbO_2_] in PMC, SMA, and left IPL in endurance group and right PMC at 20% for active control. Bilateral decrease in [HHb] in motor cortices in both groups except at 20% in active control.
Shibuya and Kuboyama (2010)	Cross-sectional	M1	Maximal voluntary static handgrip test	No differences in [HbO_2_] between groups. Increased [HbO_2_] in M1 at voluntary exhaustion compared to baseline in non-athlete group and decrease in [HHb] below baseline (*p* < 0.05). Decreased [HbO_2_] (*p* < 0.05) in M1 at voluntary exhaustion and decrease in [HHb] below baseline (*p* < 0.05) in the athlete group.
**Visual**				
Fabiani et al. (2014)	Cross-sectional	Primary visual cortex	Visual stimulation (Checkerboard reversals at different frequencies)	Positive correlation between [HbO_2_] and V̇O_2*max*_ (*p* < 0.05) in older adults. Correlation between age and [HHb] was significant (*p* < 0.05) whereas [HHb] and V̇O_2*max*_ was not.

*DLPFC, Dorsolateral prefrontal cortex; GET, Gas exchange threshold; IPL, Inferior parietal lobe; M1, Primary motor cortex; PFC, Prefrontal cortex; PMC, Premotor cortex; SMA, Supplementary motor area; SPL, Superior parietal lobe; VLPFC, Ventrolateral prefrontal cortex; VTP, Ventilatory turn point.*

Seven studies examined the effects of CRF during an exercise task, five studies evaluated the effects during a cognitive task, one study examined a cognitive-motor dual-task, and one study examined a visual task. CRF was assessed using direct and estimated measures. Ten studies used a cycle ergometer test, two studies used a treadmill test, one study used self-report questionnaires, and one study did not specify which test was used but provided a V̇O_2*max*_ cut-off score for each group (Shibuya and Kuboyama, 2010). In addition, the hemodynamic response was measured using both [HbO_2_] and [HHb] in 10 studies whereas two studies exclusively examined [HbO_2_], and two studies only examined [HHb]. These measurements were taken in the PFC across 11 studies, motor cortices in two studies, and visual cortex in one study.

### Participant Characteristics

A total of 530 participants were examined and sample sizes ranged from 11 to 66. Seven studies consisted exclusively of male participants whereas three studies only enrolled females, and four studies evaluated both males and females. The age groups being examined varied across studies including five that evaluated younger adults (18–26 years old), four examined older adults (≥60 years old), three studies compared older and younger adults, and two studies evaluated middle-aged adults (40–60 years old). Amongst the studies that compared older and younger adults, high and low levels of CRF were only established in the older adult group in two studies (Fabiani et al., 2014; Agbangla et al., 2019a). Participant characteristics for included studies are indicated in Table 2.

**TABLE 2:** Participant characteristics for included studies.

Author (Year)	Sample size (Females)	Age ± SD	Cardiorespiratory fitness test	Cardiorespiratory fitness level (mL/kg/min)
**Cognitive**				
Agbangla et al. (2019a)	Males and females YA: *n* = 19 (2F) OA LF: *n* = 16 (9F) OA HF: *n* = 21(13F)	YA: 19.1 ± 1 OA LF: 70.31 ± 4.33 OA HF: 67.90 ± 4.86	YA: Maximal fitness test (20 m shuttle run) OA: NASA/JSC physical activity scale	V̇O_2*max*_ in OA (*p* = 0.0004) YA: 54.83 ± 7.21 OA LF: 17.4 ± 6.6 OA HF: 26.1 ± 6.7
Albinet et al. (2014)	Females OA LF: *n* = 17 OA HF: *n* = 17	LF: 68.88 ± 3.87 HF: 67.32 ± 4.48	Maximal continuous graded exercise test, cycle ergometer	V̇O_2*max*_ (*p*-value not specified) LF: 20 ± 2.7 HF: 29.8 ± 6.5
Dupuy et al. (2015)	Females YA: *n* = 22 OA: *n* = 36	YA LF: 23.5 ± 5.3 YA HF: 24.5 ± 3.1 OA LF: 60.8 ± 5.6 OA HF: 63.0 ± 3.1	Maximal continuous graded exercise test, cycle ergometer	V̇O_2*max*_ (*p* < 0.05) in YA and OA groups YA LF: 36.4 ± 5.3 YA HF: 46.6 ± 7.0 OA LF: 21.4 ± 7.1 OA HF: 30.1 ± 1.5
Hyodo et al. (2016)	Males OA: *n* = 60	OA: 70.3 ± 3.2	Graded exercise test, cycle ergometer	Ventilatory threshold OA: 14.9 ± 3.8
Mekari et al. (2019)	Males and females OA: *n* = 66 (44F)	LF: 69.6 ± 4.68 HF: 66.5 ± 7.41	Maximal continuous graded exercise test, cycle ergometer	V̇O_2p*eak*_ (*p* > 0.05) LF: 18.4 ± 4.77 HF: 27.5 ± 5.92
**Dual-task**				
Goenarjo et al. (2020a)	Males YA LF: *n* = 12 YA HF: *n* = 12	HF: 22.8 ± 4.2 LF: 24.8 ± 5.1	Maximal continuous graded exercise test, treadmill	V̇O_2p*eak*_ (*p* < 0.05) LF: 36.7 ± 4.1 HF: 56.0 ± 6.7
**Exercise**				
Brugniaux et al. (2014)	Males YA sedentary: *n* = 12 YA active: *n* = 12	Sedentary: 24 ± 5 Active: 26 ± 7	Incremental exercise, cycle ergometer	V̇O_2*max*_ *(p* < 0.05) Sedentary: 33 ± 5 Active: 52 ± 9
Buzza et al. (2016)	Females Middle age STT: *n* = 13 Middle age LTT: *n* = 13	STT: 51.5 ± 5.0 LTT: 47.5 ± 5.0	Ramp incremental exercise, cycle ergometer	VTP *(p* < 0.05) STT: 20.2 ± 5.1 LTT: 29.0 ± 6.4
Buzza et al. (2020)	Males Middle age STT: *n* = 14 Middle age LTT: *n* = 14	STT: 48.6 ± 5.5 LTT: 46.1 ± 4.6	Ramp incremental exercise, cycle ergometer	GET V̇O_2_ *(p* < 0.05) STT: 26.6 ± 5.5 LTT: 37.7 ± 5.4
Caen et al. (2019)	Males YA: *n* = 11	YA: 21.8 ± 1.2	Maximal ramp incremental exercise, cycle ergometer	V̇O_2p*eak*_ (*p* < 0.05) Pre: 52.4 ± 3.5 Post: 56.4 ± 3.8
Oussaidene et al. (2015)	Males Untrained: *n* = 11 Endurance trained: *n* = 13	Endurance trained: 24 ± 6 Untrained: 26 ± 5	Ramp cycle exercise test, cycle ergometer	V̇O_2*max*_ *(p* < 0.05) Endurance trained: 61.2 ± 8.0 Untrained: 47.3 ± 4.0
Seidel et al. (2019)	Males and females Endurance trained: *n* = 22 (8F) Active control: *n* = 20 (8F)	Endurance trained: 28.82 ± 3.92 Control: 24.80 ± 3.14	Incremental cycling test, cycle ergometer	V̇O_2*max*_ (*p* < 0.001) Endurance trained: 64.59 ± 10.07 Active control: 52.20 ± 7.21
Shibuya and Kuboyama (2010)	Males Athletes: *n* = 7 Non-athletes: *n* = 7	Males Whole sample: 25.2 ± 1.4	Not specified	V̇O_2*max*_ (*p*-value not specified) Highly trained athletes: 60 Non-athletes: < 45
**Visual**				
Fabiani et al. (2014)	Males and females YA: *n* = 19 (9F) OA HF: *n* = 20 (11F) OA LF: *n* = 24 (13F)	YA: 22.3 ± 2.0 OA HF: 70.3 ± 4.2 OA LF: 72.2 ± 5.2	YA: Self-reported physical activity OA: Modified Balke protocol	V̇O_2*max*_ (*p* < 0.0001) YA: Not measured OA HF: 30.7 ± 6.7 OA LF: 18.9 ± 3.8

*GET, Gas exchange threshold; HF, High fit; LF, Low fit; LTT, Long term trained; NASA/JSC, NASA/Johnson Space Center; OA, Older adults; STT, Short term trained; VTP, Ventilatory turn point; YA, Younger adults.*

### Cardiorespiratory Fitness Terminology

In the included studies, various terms were used to describe CRF levels. More specifically, the studies evaluating CRF during exercise tasks used terms such as moderately active and sedentary (Brugniaux et al., 2014; Caen et al., 2019), short-term and long-term training (Buzza et al., 2016, 2020), trained and untrained (Oussaidene et al., 2015), endurance athlete and control (Seidel et al., 2019), and athlete and non-athlete (Shibuya and Kuboyama, 2010). In studies measuring cerebral oxygenation during cognitive tasks, the terms higher and lower fit were used to characterize CRF level.

### Effects of Cardiorespiratory Fitness During Cognitive Tasks

Two studies examined the effects of CRF level on [HbO_2_] activation during the Stroop task (Dupuy et al., 2015; Hyodo et al., 2016), one study used the n-back task (Agbangla et al., 2019a), control counting and random number generation (Albinet et al., 2014), and trail making tests part A and B (Mekari et al., 2019). Four studies measured [HbO_2_] and [HHb] (Albinet et al., 2014; Dupuy et al., 2015; Hyodo et al., 2016; Agbangla et al., 2019a) whereas one study only measured [HbO_2_] (Mekari et al., 2019). All studies measured PFC activation in older adults and divided the participants into high and low fit groups based on V̇O_2*max*_ measured by cycle ergometer tests except for one study that used a self-report questionnaire in older adults (Agbangla et al., 2019a).

#### Oxyhemoglobin During Executive Function Tasks

High fit groups demonstrated greater activation in the frontal lobe than low fit groups. More specifically, increased [HbO_2_] change was observed in the right inferior frontal gyrus and bilaterally in the dorsolateral PFC in high compared to low fit women during a Stroop task, regardless of age (Dupuy et al., 2015), and random number generation task (Albinet et al., 2014), respectively. This between groups effect was not observed during a control counting task, which was considered to be less demanding (Albinet et al., 2014). Within the high fit group, similar activation was observed in the right and left PFC whereas significantly lower [HbO_2_] was observed in the right dorsolateral PFC compared to left in the low fit group. Right dorsolateral PFC activation was also greater overall in the high fit group compared to low fit group (Albinet et al., 2014). A second study examining the effects of CRF in males during the Stroop task revealed that the higher fit group was associated with more left-lateralized dorsolateral PFC activation compared to the lower fit group (Hyodo et al., 2016).

The final two studies measured cerebral oxygenation during an n-back task (Agbangla et al., 2019a) and a trail making task (Mekari et al., 2019) in a mixed sample of older males and females. Cerebral oxygenation mediated the relationship between CRF and executive function performance on the trail making test part B (Mekari et al., 2019). In other words, increased PFC [HbO_2_] in high fit older adults resulted in better performance on part B of the trail making test than the low fit group. A similar interaction was observed during the n-back task in which PFC [HbO_2_] increased in the high compared to low fit group on the 2- and 3-back tasks resulting in better accuracy performance (Agbangla et al., 2019a). In addition, the lower fit group exhibited less [HbO_2_] activation overall compared to the high fit group.

#### Deoxyhemoglobin During Executive Function Tasks

Four studies measured the effects of CRF on [HHb] during executive functions tasks. Each study measured PFC activation but only one found a greater [HHb] decrease in high compared to low fit groups (Agbangla et al., 2019a). More specifically, this study used an n-back task in a mixed sample of female and male older adults whose CRF was assessed using a self-report questionnaire (Agbangla et al., 2019a). There were no significant differences in the other three studies that evaluated CRF using a cycle ergometer test and changes in cerebral oxygenation in the PFC (Albinet et al., 2014; Dupuy et al., 2015; Hyodo et al., 2016). In addition, these studies only examined female (Albinet et al., 2014; Dupuy et al., 2015) or male (Hyodo et al., 2016) participants during a controlled counting or random number generation task (Albinet et al., 2014) or Stroop task (Dupuy et al., 2015; Hyodo et al., 2016).

#### Oxyhemoglobin and Deoxyhemoglobin During Visual Tasks

One study measured the effects of CRF level on [HbO_2_] and [HHb] in older adults (Fabiani et al., 2014). By measuring cerebral oxygenation changes in the visual cortex, findings revealed a significant positive correlation between [HbO_2_] and V̇O_2*max*_ whereby low fit older adults demonstrated reduced [HbO2] activation compared to the high fit group. There was no correlation, however, between [HHb] and V̇O_2*max*_.

#### Oxyhemoglobin and Deoxyhemoglobin During Cognitive-Motor Dual-Tasks

One study measured the effects of CRF level on [HbO_2_] and [HHb] during a dual-task (Goenarjo et al., 2020b). The dual-task was composed of an auditory n-back task and walking and there were no significant differences in [HbO_2_] in either PFC hemisphere and between high and low fit younger adults. However, there was a significantly greater decrease in [HHb] in the high compared to low fit group in the right and left PFC during dual-tasks and a negative correlation between V̇O_2p*eak*_ and [HHb].

### Effects of Cardiorespiratory Fitness During Exercise Tasks

#### Oxyhemoglobin During Acute Exercise Tasks

Oxyhemoglobin was measured across acute bouts of exercise that included cycle ergometer tests and incremental cycling (Brugniaux et al., 2014), maximal and submaximal ramp exercises (Oussaidene et al., 2015), cycling with a constant load (Seidel et al., 2019), and a maximal voluntary static handgrip task (Shibuya and Kuboyama, 2010). During acute exercise, PFC activation differed between high and low fit groups (Brugniaux et al., 2014; Oussaidene et al., 2015). There were progressive [HbO2] increases during incremental cycling in the moderately active group until 80% of V̇O_2*max*_ after which [HbO2] leveled off and declined (Brugniaux et al., 2014). [HbO_2_] remained constant despite increased exercise intensity in the low fit group. At submaximal exercise intensity, [HbO_2_] was higher in an endurance trained compared to untrained younger adults (Oussaidene et al., 2015). The maximum cerebral oxygenation threshold was also higher in the trained group, but the threshold occurred at a similar V̇O_2*max*_ in both groups.

Cerebral oxygenation of the parietal lobe and motor cortices including the primary motor cortex (M1), supplementary motor area (SMA), and premotor cortex (PMC) were measured during a maximal voluntary static handgrip test (Shibuya and Kuboyama, 2010), and a cycle ergometer test (Seidel et al., 2019). There were no [HbO_2_] and CRF level interactions during the cycling test but within the endurance trained group, there was a decrease in [HbO_2_] in the PMC, SMA, and left inferior parietal lobe (Seidel et al., 2019). In the active control group, this effect was only observed in the right-hemispheric PMC at 20% intensity (Seidel et al., 2019). During the handgrip exercise test, there was continued activation in the contralateral M1 in the non-athlete group at voluntary exhaustion compared to M1 activation that dropped below baseline values in the athlete group (Shibuya and Kuboyama, 2010).

#### Deoxyhemoglobin During Acute Exercise Tasks

Two studies, one examining male and the other examining female older adults, measured deoxyhemoglobin during a ramp incremental test at 25, 80, or 90% intensity or square wave constant load test at 90% or peak intensity (Buzza et al., 2016, 2020). In the study examining females, there were no significant interactions between CRF level and PFC activation at 25, 80, and 90% or peak intensity in short-term (6–24 months) or long-term (>5 years) groups (Buzza et al., 2016). However, the study examining males revealed greater [HHb] changes in the PFC during the square wave constant load test in the long compared to short-term trained group at 90% intensity (Buzza et al., 2020). In a study measuring left PFC activation, there were no differences in [HHb] between endurance-trained and untrained young males (Oussaidene et al., 2015).

Two studies measured changes in [HHb] in the motor cortex. The first study did not find significant interactions between endurance-trained athletes and active controls during a cycling test (Seidel et al., 2019). There was, however, a larger [HHb] decrease at 60% compared to 20% intensity across all participants in the left PMC. A second study evaluated the effect of a static handgrip exercise test on contralateral M1 activation in athlete and non-athlete younger adults (Shibuya and Kuboyama, 2010). Findings revealed that [HHb] activation decreased after 20 s of exercise and continued below baseline values from 30 s to exhaustion in the non-athlete group. The athlete group demonstrated lower [HHb] levels than baseline values at 30 s to exhaustion.

#### Oxyhemoglobin and Deoxyhemoglobin Following a Training Intervention

Cerebral oxygenation was measured during a maximal incremental test before and after a 6-week cycling training intervention (Caen et al., 2019). Findings revealed that after an aerobic training intervention, participants who displayed greater V̇O_2*max*_ had a higher total [HbO_2_] and total hemoglobin amplitude during maximal exercise compared to pre-training (Caen et al., 2019).

### Functional Near-Infrared Spectroscopy Processing and Analysis Methods

FNIRS recordings during exercise tasks lasted between 2 and 32 min, which was substantially longer than studies using cognitive tasks where brain activity was recorded for 30–150 s. Physiological and/or motion filters were used to remove artifacts from noisy data in all studies evaluating cerebral oxygenation during cognitive and visual tasks. In contrast, the study evaluating dual-tasks stated that they did not use any filters but artifacts were identified through visual inspection and replaced by interpolation of adjacent data (Goenarjo et al., 2020a). Of the studies examining exercise tasks, filtering methods were not specified except for one study that used short-distance channels to eliminate physiological artifacts (Seidel et al., 2019).

All but one study used continuous wave fNIRS, which are the most commercially available devices (Scholkmann et al., 2014). Fabiani et al. (2014) used a frequency domain device. Accordingly, thirteen studies measured relative changes in cerebral oxygenation by subtracting baseline values from task-evoked changes. A linear regression approach was used by one study to assign a slope coefficient to [HbO_2_] and [HHb] for the entire response signal (Albinet et al., 2014). Channel configuration varied between studies with four using three channels (Buzza et al., 2016, 2020; Mekari et al., 2019; Goenarjo et al., 2020b), three studies using two (Shibuya and Kuboyama, 2010; Albinet et al., 2014; Caen et al., 2019) or eight channels (Brugniaux et al., 2014; Oussaidene et al., 2015; Agbangla et al., 2019a), and one study using 16 (Dupuy et al., 2015), 22 (Seidel et al., 2019), 32 (Fabiani et al., 2014), or 48 (Hyodo et al., 2016) channels, respectively. Across all studies, the lower wavelength ranged from 690 to 794 nm and the upper wavelength ranged from 830 to 905 nm, which was largely dependent on the fNIRS device.

### Differences Between Cardiorespiratory Fitness Measures

CRF was measured using direct measures of V̇O_2*max*_, V̇O_2p*eak*_, ventilatory threshold, or peak power output in 11 studies and self-report questionnaires in three studies. The study using a ventilatory threshold justified using this measure because it was more convenient for older adults (Hyodo et al., 2016). Amongst the studies using direct measures, four studies divided the participants into high and low fit groups based on a median split or by excluding the middle group of CRF scores and analyzing the upper and lower thirds (Albinet et al., 2014; Fabiani et al., 2014; Mekari et al., 2019; Goenarjo et al., 2020a). The remaining studies divided groups based on published age and gender norms of CRF. In addition to direct measures of CRF, five studies assessed whether participants had a background in different types of physical activity or training during the recruitment stage (Albinet et al., 2014; Brugniaux et al., 2014; Oussaidene et al., 2015; Caen et al., 2019; Seidel et al., 2019). This included a 7-point physical activity rating used to estimate V̇O_2*max*_ (Albinet et al., 2014), a self-report physical activity questionnaire (Brugniaux et al., 2014), a simple question about past involvement in recreational sports (Caen et al., 2019), or questions that determined the number of hours per week of physical activity (Oussaidene et al., 2015; Seidel et al., 2019).

The three studies using self-report questionnaires to determine CRF consisted of the NASA/Johnson Space Center physical activity questionnaire, which required older adults to rate their physical activity on a scale from 0 to 7 and was adjusted for their age, body mass index (BMI), and sex (Agbangla et al., 2019a). In comparison, two studies used self-reported physical activity logs to track training minutes of moderate to vigorous exercise per week (Buzza et al., 2016, 2020).

### Assessment of Risk of Bias

Based on the JBI quality assessment checklist, each of the 14 studies was of sufficient quality to be included in this review (Table 3). In all studies, the exposure was clearly described, the outcomes were clearly defined, and statistical analyses were appropriately chosen. All studies described the participant characteristics in sufficient detail, but two studies did not clearly describe the setting. Despite this, it can be assumed that these studies were conducted in a university lab setting due to the specialized equipment involved in CRF testing. Objective criteria were used to measure CRF in 13 studies whereas one study indicated the cut-offs between high and low groups but did not indicate what test was used to measure V̇O_2*max*_ in each group (Shibuya and Kuboyama, 2010). Lastly, seven studies identified and controlled for confounding variables such as education level. Five studies did not control for confounding variables, and two studies were unclear. These studies were not excluded because they reported confounding characteristics in a table format, which were not significantly different between groups. Therefore, it is unlikely that they contributed to sources of bias between studies.

**TABLE 3:** JBI quality assessment.

	Criteria							
Author (Year)	1	2	3	4	5	6	7	8
Agbangla et al. (2019a)	Yes	Yes	Yes	Yes	Yes	Yes	Yes	Yes
Albinet et al. (2014)	Yes	Yes	Yes	Yes	Yes	Yes	Yes	Yes
Brugniaux et al. (2014)	Yes	Unclear	Yes	Yes	No	No	Yes	Yes
Buzza et al. (2016)	Yes	Yes	Yes	Yes	Yes	Yes	Yes	Yes
Buzza et al. (2020)	Yes	Unclear	Yes	Yes	Yes	Yes	Yes	Yes
Caen et al. (2019)	Unclear	Yes	Yes	Yes	No	No	Yes	Yes
Dupuy et al. (2015)	Yes	Yes	Yes	Yes	No	No	Yes	Yes
Fabiani et al. (2014)	Yes	Yes	Yes	Yes	Unclear	Unclear	Yes	Yes
Goenarjo et al. (2020a)	Yes	Yes	Yes	Yes	Yes	Yes	Yes	Yes
Hyodo et al. (2016)	Yes	Yes	Yes	Yes	Yes	Yes	Yes	Yes
Mekari et al. (2019)	Yes	Yes	Yes	Yes	Yes	Yes	Yes	Yes
Oussaidene et al. (2015)	Yes	Yes	Yes	Yes	No	No	Yes	Yes
Seidel et al. (2019)	Yes	Yes	Yes	No	Unclear	Unclear	Yes	Yes
Shibuya and Kuboyama (2010)	Yes	Yes	Yes	Unclear	No	No	Yes	Yes

*1. Were the criteria for inclusion in the sample clearly defined? 2. Were the study subjects and the setting described in detail? 3. Was the exposure measured in a valid and reliable way? 4. Were objective, standard criteria used for measurement of the condition? 5. Were confounding factors identified? 6. Were strategies to deal with confounding factors stated? 7. Were the outcomes measured in a valid and reliable way? 8. Was appropriate statistical analysis used?*

## Discussion

### Effects of Cardiorespiratory Fitness on Cerebral Oxygenation During Cognitive Tasks

Previous reviews have outlined the effects of chronic exercise on cognitive performance (Li et al., 2017; Rathore and Lom, 2017), but few have assessed the impact of CRF on cerebral oxygenation. In a meta-analysis, Rooks et al. (2010) examined the impact of training status on cerebral oxygenation during incremental tests without considering how CRF and training status were measured (i.e., V̇O_2*max*_ or physical activity level). More recently, Agbangla et al. (2021) reviewed the impact of CRF on cerebral oxygenation during cognitive tasks. This review examined the effects of CRF level, as determined by direct or estimated measures, on cerebral oxygenation during exercise and cognitive task performance. Exercise-related effects on cognition have been explored using the cardiorespiratory hypothesis, which maintains that improvements in cognitive performance are moderated by factors such as increased cerebral perfusion in individuals with greater CRF (Agbangla et al., 2019b).

Studies examining the PFC revealed increased [HbO_2_] in higher compared to lower fit groups during cognitive tasks. In fact, most studies in this review used tasks that draw on executive functions since they are susceptible to age-related declines and can be improved with exercise interventions (Colcombe and Kramer, 2003). There is evidence that the frontal lobe is disproportionately affected by aging but is activated during executive function tasks (West, 1996). In older adults, this results in bilateral PFC activation compared to younger adults who demonstrate unilateral activation to support task performance (Reuter-Lorenz and Cappell, 2008). This is outlined in the Compensation-Related Utilization of Neural Circuits Hypothesis (CRUNCH) which accounts for the effect of task demands and processing capacity that when exceeded, results in performance declines (Reuter-Lorenz and Cappell, 2008). Therefore, PFC activation is more pronounced with increasing executive function demands and greater CRF can facilitate these processes (Albinet et al., 2014; Agbangla et al., 2019a; Goenarjo et al., 2020a). In the case of M1, activation is observed in the contralateral hemisphere to the movement being performed (Shibuya and Kuboyama, 2010). Previous studies have reported that right-handed individuals activate the left M1 but recruit M1 bilaterally during exhaustive exercise to compensate for decreased muscle force (Shibuya et al., 2008). Therefore, both the PFC and M1 can demonstrate bilateral activation as a mechanism of compensation.

In studies comparing younger and older adults, higher V̇O_2*max*_ only contributed to increased cerebral oxygenation in older adults (Dupuy et al., 2015; Agbangla et al., 2019a). In other words, high and low fit younger adults (Dupuy et al., 2015) as well as a pooled group (Agbangla et al., 2019a) did not exhibit increased cerebral oxygenation or differences in performance. These findings have been attributed to the high functioning status of young adults and intact brain structures that help maintain cognitive performance regardless of CRF level. In addition, older adults aged 65 and over have shown the greatest capacity to demonstrate improvements in executive function compared to younger adults (Colcombe and Kramer, 2003). Therefore, differences in CRF may only lead to small improvements in cerebral oxygenation, which may be less evident in younger compared to older adults.

[HHb] findings prove to be more variable with studies reporting both decreased and insignificant changes based on CRF. Two studies found decreased [HHb] during executive function and dual-tasks in high compared to low fit younger and older adults (Agbangla et al., 2019a; Goenarjo et al., 2020b). In contrast, one study indicated that the lack of significant findings was due to their fNIRS device, which was not configured to measure [HHb] (Hyodo et al., 2016). Insignificant differences in [HHb] can also be attributed to its low signal to noise ratio making it hard to identify the hemodynamic component of the signal. Nonetheless, [HHb] is less likely to be influenced by systemic and motion artifacts making it a reliable measure during tasks that require unrestricted movements (Menant et al., 2020). To better interpret these results, it is important to remember that during cognitive stimulation, the fNIRS responses are manifested by an increase in [HbO_2_] and a decrease in [HHb] (Villringer, 1997). [HHb] is also strongly correlated with brain activity and inversely correlated with the BOLD signal (Villringer, 1997). The greater the decrease in [HHb], the more the BOLD signal increases. Based on this information, a greater decrease in [HHb] during a cognitive task in high fit subjects could represent greater brain activity as shown by Colcombe et al. (2004).

Studies examining mixed samples of male and female participants demonstrated that increased CRF was associated with increased cerebral oxygenation and better performance (Agbangla et al., 2019a; Mekari et al., 2019). There are, however, known differences in CRF between males and females that contribute to changes in cerebral oxygenation (Colcombe and Kramer, 2003; Erickson et al., 2007; Dimech et al., 2019). For example, findings differed across male and female groups in the present review such that high fit females demonstrated increased bilateral activation in the PFC (Albinet et al., 2014) and increased right activation as task demands increased (Dupuy et al., 2015). Older males, however, only demonstrated left lateralized PFC activation in the higher fit group, which is a pattern typically observed in younger adults (Hyodo et al., 2016). This is outlined in the hemispheric asymmetry reduction in older adults (HAROLD) model whereby bilateral activation is expected due to decreased white matter integrity, vascularization, and less efficient adaptations to task-related metabolic demands (Cabeza, 2002; Hyodo et al., 2016; Agbangla et al., 2019b). Upon re-examining the studies investigating mixed samples, participants were predominantly female. Therefore, data from female participants may be contributing to bilateral activation in mixed samples more so than males. More evidence is needed that directly compares activation in males and females across different CRF levels.

### Effect of Cardiorespiratory Fitness on Cerebral Oxygenation During Exercise

Incremental and maximal exercise elicit an increased metabolic demand for oxygen. In the present review, cerebral oxygenation was measured during acute cycling exercise and in one case during a static handgrip task (Shibuya and Kuboyama, 2010). During submaximal exercise, [HbO_2_] was greater in the PFC of trained compared to untrained young males (Oussaidene et al., 2015). In addition, moderately active young males demonstrated increased PFC oxygenation until 80% V̇O_2*max*_ compared to the sedentary group, which displayed constant but lower cerebral oxygenation (Brugniaux et al., 2014). These findings build on a previous meta-analysis where healthy participants demonstrated steady increases in cerebral oxygenation during incremental exercise compared to their baseline resting levels (Rooks et al., 2010).

In terms of [HHb], two studies measured middle-aged females and males, respectively (Buzza et al., 2016, 2020). Only the male long-term training group (higher fit) demonstrated greater PFC [HHb] compared to the short-term training group (lower fit) at 90% intensity on a cycle ergometer (Buzza et al., 2020). A similar interaction was observed in younger adults whereby left PFC [HHb] increased in a trained vs. untrained group (Oussaidene et al., 2015). From low to submaximal intensities, PFC [HbO_2_] is expected to increase until it reaches a plateau near maximum intensity (Jung et al., 2015). During the handgrip exercise test, however, activation in M1, which is responsible for sending efferent information to the contracting hand muscles, continued to rise in the non-athlete group at voluntary exhaustion whereas it dropped below baseline values in the athlete group (Shibuya and Kuboyama, 2010). More specifically, participants were right-handed and cerebral oxygenation was measured in the contralateral M1. Continued activation may be a mechanism used to compensate for decreased muscle force when exhausted (Shibuya et al., 2008). A second mechanism is the bilateral activation of M1, which has been observed during low intensity static handgrip tasks (Shibuya et al., 2008). For the same task, compensation and greater oxygenation in the lower compared to higher fit group reflects how [HbO_2_] and [HHb] are regulated in M1 during exhaustive motor tasks (Shibuya and Kuboyama, 2010).

### Effects of Exercise Training on Cerebral Oxygenation

Numerous studies have demonstrated that aerobic training is beneficial for cognitive performance, but few interventional studies have evaluated the effect of aerobic training on brain oxygenation during physical exercise or cognitive tasks. Only one study in this review measured a 6-week aerobic exercise training intervention and demonstrated that [HbO_2_] and [HHb] amplitude and slope increased post-intervention in younger adults during maximal incremental exercise test (Caen et al., 2019). It is unclear if this is the case for older adults, but previous reports have demonstrated improvements in executive functions following exercise interventions (Voss et al., 2012; Northey et al., 2018; Domingos et al., 2021). In addition, fitness training selectively improved executive control processes compared to speed, controlled, and spatial ability in older adults (Colcombe and Kramer, 2003). Fewer studies have examined cerebral oxygenation following exercise interventions but changes in cerebral blood flow have been observed in cortical and subcortical regions (Brown et al., 2010; Chapman, 2013). To the best of our knowledge, only Coetsee and Terblanche (2017) reported lower cerebral oxygenation in older adults after aerobic training, but the measure of V̇O_2*max*_ was not explicitly stated. In addition, the meta-analysis by Rooks et al. (2010) reported greater cerebral oxygenation during incremental exercise tests between trained and untrained participants but lacked defined criteria for training status making the results difficult to compare. Previous reports have used direct measures of CRF in older adults (Dupuy et al., 2015) while others suggest that estimated measures are more feasible to obtain in older adults (Agbangla et al., 2019a). Nonetheless, more training interventions are needed to assess the effects of CRF on cerebral oxygenation.

### Possible Mechanisms Underlying Improved Cerebral Oxygenation With Higher Cardiorespiratory Fitness

Exercise has known benefits on cerebrovascular health such that the cardiovascular system is involved in delivering oxygen and regulating cerebral metabolism to sustain cognitive processing. From a cognitive perspective, exercise may increase angiogenesis, neurovascular plasticity, and oxygen saturation in brain regions related to cognitive performance including prefrontal and motor cortices and the hippocampus (Stimpson et al., 2018). Exercise also upregulates growth factors like brain-derived neurotrophic factor (BDNF), insulin-like growth factor (IGF), and vascular endothelial growth factor (VEGF), which are involved in synaptic plasticity, neurogenesis, promote angiogenesis, and support memory (Cotman and Berchtold, 2002; Ploughman, 2008; Davenport et al., 2012; Hayes et al., 2013). These neurotrophins can also offset the effects of age-related cerebral atrophy that interfere with adequate oxygen delivery to the brain (Ainslie et al., 2008; Erickson et al., 2014). For example, 3 months of aerobic exercise have been reported to increase neurotrophins and cerebral blood volume (Pereira et al., 2007). Therefore, higher fit individuals demonstrate increased BDNF compared to lower fit individuals because of improved cerebral blood flow and better vascularization, which foster neurotrophic and growth factors in the brain (Brown et al., 2010; Stimpson et al., 2018). In addition, older adults who are more physically active have been found to display a higher number of small cerebral vessels than less physically active older adults (Bullitt et al., 2009). Angiogenesis is upregulated by VEGF which also promotes endothelial cell proliferation (Cotman and Berchtold, 2002). Similarly, exercise increases the production of plasmatic VEGF which is the main growth factor associated with capillary formation in the brain (Cotman and Berchtold, 2002; Duman, 2005). Stimpson et al. (2018) and Dupuy et al. (2019) summarized these factors in a simplified model that describes the relationship between physical activity and cognition. In combination with these molecular mechanisms, it has also been observed that higher fit individuals have higher cerebral blood flow at rest, and during the tilt test and exercise than lower fit people (Murrell et al., 2011a,b). This explains the reports of greater cerebral oxygenation in higher fit individuals. Given that higher CRF is associated with increased cerebral oxygenation, fNIRS measures can complement the existing literature on the mechanisms involved in exercise and improved cognition.

### Functional Near-Infrared Spectroscopy Devices and Processing Variability

Given the growing popularity of fNIRS in cognitive and exercise physiology research, guidelines have been established to increase reporting transparency and the reproducibility of different study designs (Menant et al., 2020; Yücel et al., 2021). In terms of fNIRS devices, most commercially available continuous wave fNIRS systems have the capability of measuring [HbO_2_] and [HHb], which is an advantage over fMRI. Therefore, studies should report on both measures to broaden our understanding of potential shifts between increases and decreases in these measures related to CRF. Amongst the studies in this review, the most common justification for only reporting [HbO_2_] is that it better reflects cortical activation, but it should be noted that it contains a larger signal-to-noise ratio (Strangman et al., 2002). In terms of [HHb], it is less contaminated by systemic artifacts, but the signal is attenuated compared to [HbO_2_], and certain devices are not configured to assess both measures (Kirilina et al., 2012; Hyodo et al., 2016).

As stated in the guidelines and consensus measures by Menant et al. (2020) and Yücel et al. (2021), signal processing methods should be clearly described. Many studies in this review were unclear or did not specify the methods used to process motion artifacts. For example, cycling tasks are expected to produce greater motion artifacts than seated cognitive tasks due to increased movement (Yücel et al., 2021). Removing irrelevant artifacts from the signal can be achieved through filtering, visual inspection, and is sometimes overcome with specific instructions for participants to minimize sudden head movements (Menant et al., 2020). In order to adequately compare and contrast different research studies and to fully understand cerebral oxygenation changes associated with CRF level, detailed reporting is imperative.

The duration of fNIRS measurements also differed between cognitive and exercise tasks. Exercise measures were significantly longer than cognitive tasks given the nature of incremental cycling. In both cases, the segment analyzed was of sufficient duration to capture the hemodynamic response. Longer measures, however, could increase participant discomfort and affect brain activation due to drifts in the signal (Menant et al., 2020).

### Limitations

This systematic review included cross-sectional studies with relatively small sample sizes. In addition, only one study examined the effects of exercise training on cerebral oxygenation. Future studies should consider implementing randomized controlled trials to ensure a comprehensive examination and minimal bias is introduced when examining the effects of CRF on cerebral oxygenation. The self-reported questionnaires to estimate V̇O_2*max*_ may have introduced biased responses if participants overestimated their physical activity levels. However, the questionnaires were reliable and a more feasible method to measure CRF in older adults. V̇O_2*max*_ also significantly differed between high and low fit groups in these studies.

Continuous wave fNIRS devices are most frequently reported in the literature, but only provide relative changes in cerebral oxygenation rather than absolute values. This limitation can be overcome by frequency and time domain fNIRS devices, which come at a greater cost, but allow for more in-depth measures. Although it is also limited to surface cortex measurements, changes in PFC and motor cortex activation were identified between studies. The interaction between these two regions should be further examined as connectivity between the PFC and motor regions is important for exercise load management, coordination, and preparation of motor movements, which may differ between high and low fit groups (Voss et al., 2016). In addition, similar deoxygenation between PFC and motor cortices has been reported during maximum intensity exercise (Subudhi et al., 2009). Lastly, preprocessing is essential for removing physiological (e.g., systemic) and motion artifacts (e.g., head movements) in the fNIRS signal. Due to the nature of physical exercise, these variables may be especially prominent. Future studies should report all preprocessing steps to increase signal quality and methodological reproducibility.

## Conclusion

CRF can affect cerebral oxygenation in young, middle-aged, and older adults. Due to the widespread applications of fNIRS in both aerobic exercise and cognitive tasks, careful attention should be placed on reporting detailed processing methods to increase study reproducibility. Nonetheless, increased CRF was generally associated with increased cerebral oxygenation in the prefrontal and motor cortices. Lastly, this review predominantly featured cross-sectional studies. Future research should attempt to reproduce these effects using fNIRS in a randomized controlled trial to identify whether these findings hold true across larger samples, populations, and training interventions.

## Author Contributions

TS developed the search with a Health Sciences librarian, translated the search for the different databases, conducted the search, screened and extracted the articles, wrote up the findings, and drafted the manuscript. OD assisted with the screening of the articles, reviewed the findings, and participated in the write-up of the manuscript. SF assisted with the screening and extraction of the articles, reviewed the findings, and participated in the write-up of the manuscript. All authors contributed to the article and approved the submitted version.

## Conflict of Interest

The authors declare that the research was conducted in the absence of any commercial or financial relationships that could be construed as a potential conflict of interest.

## Publisher’s Note

All claims expressed in this article are solely those of the authors and do not necessarily represent those of their affiliated organizations, or those of the publisher, the editors and the reviewers. Any product that may be evaluated in this article, or claim that may be made by its manufacturer, is not guaranteed or endorsed by the publisher.
