# Supplementary material for: Effects of Cardiorespiratory Fitness on Cerebral Oxygenation in Healthy Adults: A Systematic Review
Source: Front Physiol. 2022 Mar 4;13:838450. doi: 10.3389/fphys.2022.838450 (PMC8931490; doi:10.3389/fphys.2022.838450)
Supplement: Supplementary file 1 [file Table_1.DOCX]

Supplementary Material

Supplementary Table 1. Sample Medline search strategy.

| 1. | Spectroscopy, Near-Infrared/ |
| --- | --- |
| 2. | (fNIRS or NIRS or near-infrared spectroscopy).ti,ab,kf. |
| 3. | (HbO or HbO2 or O2Hb or oxyHb or oxy-Hb or oxygenated h?emoglobin or oxyh?emoglobin).ti,ab,kf. |
| 4. | (HbR or dHb or HHb or deoxyHb or deoxy-Hb or deoxygenated h?emoglobin or deoxyh?emoglobin).ti,ab,kf. |
| 5. | (HbT or THb or Hbdiff or totHb).ti,ab,kf. |
| 6. | (H?emodynamic adj2 (response* or activation* or change* or correlate*)).ti,ab,kf. |
| 7. | ((Cerebral or cortex or brain) adj2 (oxygenation or activation)).ti,ab,kf. |
| 8. | Cardiorespiratory fitness/ |
| 9. | ((Physical activity or fitness) adj2 (level* or capacity)).ti,ab,kf. |
| 10. | (VO2max or VO2peak or (max* oxygen adj2 (consumption or uptake)) or (oxygen consumption adj2 (volume or test*))).ti,ab,kf. |
| 11. | ((Cardiorespiratory or cardio-respiratory or cardiopulmonary or cardio-pulmonary or aerobic) adj2 (fitness or capacity or test*)).ti,ab,kf. |
| 12. | 1 or 2 or 3 or 4 or 5 or 6 or 7 |
| 13. | 8 or 9 or 10 or 11 |
| 14. | 12 and 13 |
